# The use of cinacalcet hinders the diagnosis of parathyroid carcinoma in a chronic dialysis patient: a case report

**DOI:** 10.1186/s12882-017-0733-0

**Published:** 2017-10-18

**Authors:** Daisuke Takada, Tatsuo Tsukamoto, Miho Fuse, Shinpei Kada, Motoko Yanagita

**Affiliations:** 1 Department of Nephrology, Kyoto University Graduate School of Medicine, 54 Shogoin Kawahara-cho, Sakyo, Kyoto, Japan; 2 Department of Nephrology & Dialysis, Kitano Hospital, The Tazuke Kofukai Medical Research Institute, Osaka, Japan; 3 Department of Otorhinolaryngology, Kyoto University Graduate School of Medicine, 54 Shogoin-Kawahara-cho, Sakyo-ku, Kyoto, 606-8507 Japan

**Keywords:** Cinacalcet, Parathyroid carcinoma, Hemodialysis, Secondary hyperparathyroidism, Ectopic calcification, Case report

## Abstract

**Background:**

Secondary hyperparathyroidism (SHPT) is a common complication in patients receiving chronic dialysis therapy. Although cinacalcet can control parathyroid function and bone turnover, preventing ectopic calcification remains challenging. Cinacalcet can also suppress PTH secretion due to parathyroid carcinoma in the same way as it does for parathyroid hyperplasia in the uremic condition. We present a case of parathyroid carcinoma partially controlled by cinacalcet, in which tumorous calcinosis was successfully resolved by total parathyroidectomy.

**Case presentation:**

A female patient in her forties who had received dialysis for 12 years was referred to our hospital for painful ectopic calcifications on her right hip joint and both knees. Although she had been treated with alfacalcidol and cinacalcet for 2 years, this therapy had been discontinued 6 months earlier as a result of hypercalcemia. The patient exhibited normocalcemia (2.37 mmol/L) and hyperphosphatemia (2.42 mmol/L) with elevated intact parathyroid hormone (707,000 μg/L). Ultrasonography revealed an enlarged parathyroid gland on the left lower side of the thyroid gland. The otolaryngologist surgeons had to perform an en bloc excision to remove this parathyroid gland because of tight adhesions. Histological examination revealed that parathyroid cells had invaded the surrounding skeletal muscle through fibrous capsules, consistent with parathyroid carcinoma. Her joint pain disappeared 2 weeks after parathyroidectomy, and the tumorous calcinosis had largely resolved after 1 year.

**Conclusions:**

Parathyroid carcinoma is a rare cause of hyperparathyroidism in end-stage kidney disease. Our case indicates that the use of cinacalcet hinders the diagnosis of parathyroid carcinoma in a chronic dialysis patient. When uncontrolled hypercalcemia and/or hyperphosphathemia develop during cinacalcet administration, parathyroidectomy should be considered to prevent a vicious exacerbation of ectopic calcification.

## Background

Secondary hyperparathyroidism (SHPT) is a common complication in patients undergoing chronic dialysis therapy. A recent medical advance has enabled suppression of the parathyroid function using cinacalcet (a calcimimetic drug). Combined with vitamin D, cinacalcet has successfully controls parathyroid function and bone turnover in many SHPT patients. Nevertheless, preventing ectopic calcification, such as tumorous calcinosis and vascular calcification, remains a challenge [1]. These two conditions can be caused by an excess load of calcium and phosphate into the soft tissue, including the blood vessels associated with a trans-differentiation of vascular smooth muscle cells and fibroblasts to the osteoblastic phenotype in the uremic condition [2, 3]. Since a non-calcium-containing phosphate binder can suppress the progression of calcification, ensuring a negative balance of calcium and phosphate in the body to reduce the load into the soft tissue is a reasonable therapeutic strategy [4, 5]. There remains a limitation in the cinacalcet and vitamin D combination therapy to control SHPT, however, especially in cases with severe ectopic calcification. Parathyroidectomy (PTx) could be considered the final choice to resolve such issues [6, 7].

Parathyroid carcinoma is a rare cause of hyperparathyroidism [8]. The use of cinacalcet to treat hypercalcemia due to parathyroid carcinoma has recently been reported [9]. Here we present a case of parathyroid carcinoma partially controlled by cinacalcet, in which the tumorous calcinosis was successfully resolved by total PTx.

## Case presentation

**Fig. 1:**
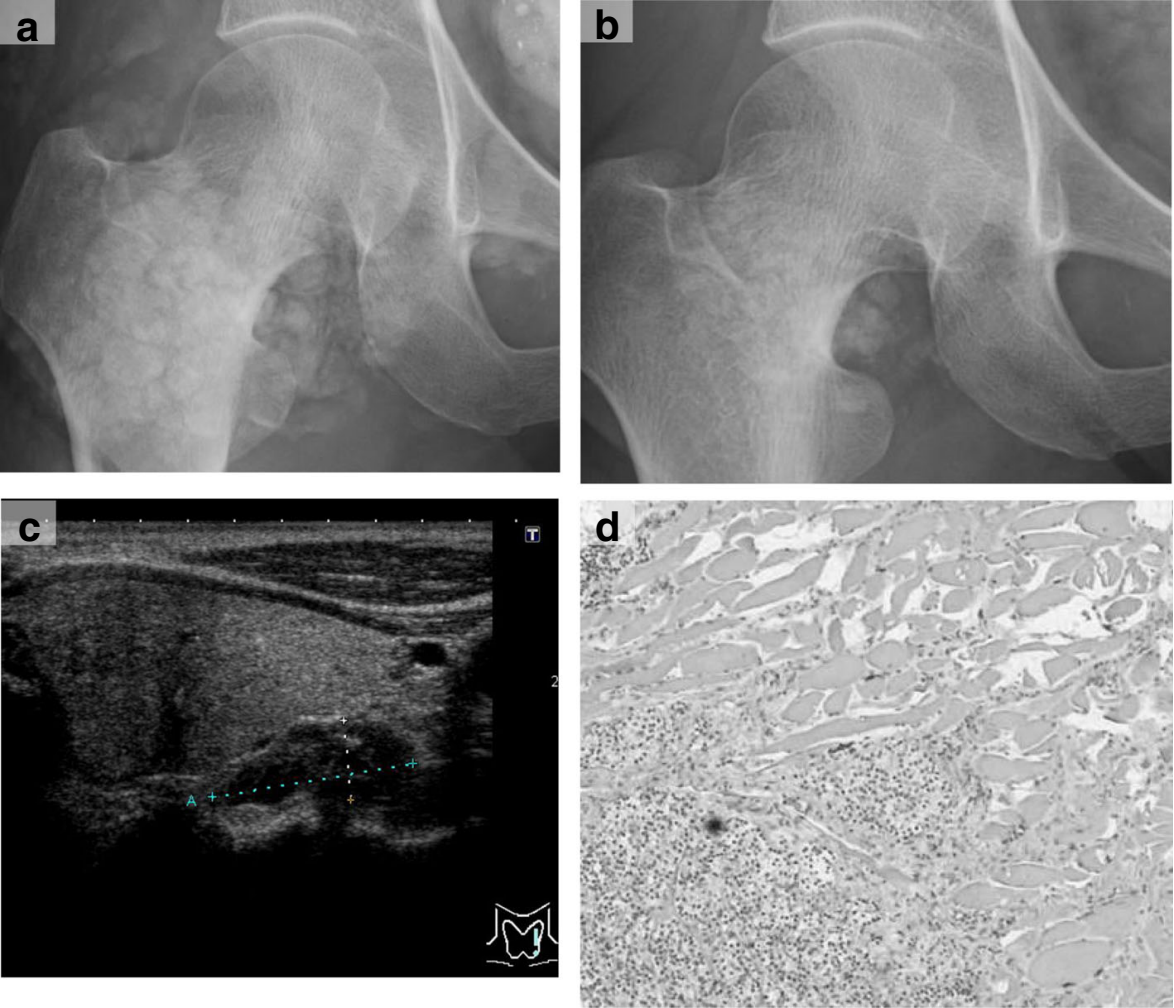
Images. Panel **a** and **b**; X-ray images of the right hip joint before (**a**) and after 6 months of total parathyroidectomy (**b**). Note that the massive calcifications around the right hip joint mostly disappeared after parathyroidectomy. Panel **c**; An enlarged parathyroid gland at the left lower side of the thyroid with a long axis of 21 mm, near the muscle layer of the esophagus. Panel **d**; Structural atypia in histological findings of the resected parathyroid gland at low (×40) magnification

**Fig. 2:**
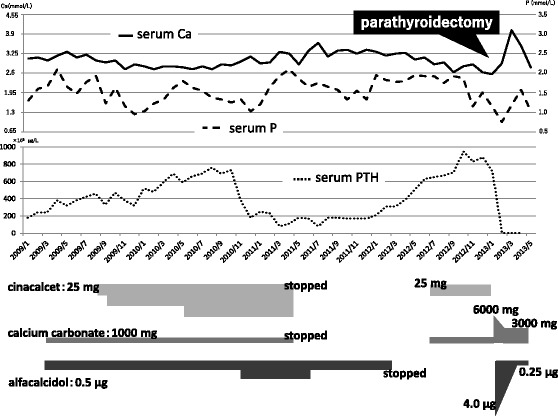
Clinical course of laboratory data (serum calcium, phosphorus, and intact-parathyroid hormone) and medication before and after parathyroidectomy

A female patient in her forties who had received regular hemodialysis (4 h, 3 times a week) for 12 years with good adherence was referred to our hospital for multiple ectopic calcifications. The cause of her end-stage kidney disease was chronic glomerulonephritis (membranoproliferative glomerulonephritis). For 2 months she had experienced progressive pain in her right hip joint and left knee while walking. X-ray examinations revealed massive calcifications in these regions (Fig. 1a). Although her SHPT was well controlled with an adequate intact parathyroid hormone (iPTH) level, her calcium level had gradually increased (Fig. 2). Although she had been treated daily with 0.5 μg alfacalcidol and 50 mg cinacalcet for 2 years, this treatment had been discontinued 6 months earlier due to hypercalcemia and hyperphosphatemia. Calcium carbonate and lanthanum carbonate were used as a phosphate binder. Even after the calcium carbonate was discontinued, she remained hypercalcemic and eventually developed joint pain. Her iPTH level increased another 500,000 μg/L before the referral. She also had unstable angina, and had received percutaneous coronary intervention with stenting for noticeably calcified coronary arteries. She had also been prescribed telmisartan, trandopril, sodium polystyrene sulfonate, aspirin, clopidogrel sulfate, eicosapentaenoic acid, omeprazole, and pravastatin.

On admission, the patient was 158.5 cm tall and weighed 49 kg. Her blood pressure was 144/88 mmHg. She had no palpable neck masses. Laboratory findings were as follows: calcium (Ca) 2.22 mmol/L; inorganic phosphate (iP) 2.42 mg/dL; alkaline phosphatase (ALP) 248 U/L; and iPTH 707,000 μg/L (Table 1). Ultrasonography of the neck showed an enlarged parathyroid gland on the left lower side of the thyroid with a long axis of 21 mm. No other parathyroid glands were identified by ultrasonography (Fig. 1c). Computed tomography of the chest detected no ectopic parathyroid in the mediastinum or lung.

**Table 1:** Laboratory data on admission

Complete blood count		
White blood cell	8600	/μL
Red blood cell	403 × 10^4^	/μL
Hemoglobin	102	g/L
Platelet	27.1 × 10^4^	/μL
Blood chemistry		
Aspartate aminotransferase	13	IU/L
Alanine aminotransferase	5	IU/L
Lactate dehydrogenase	188	IU/L
Alkaline phosphatase.	284	IU/L
γ-glutamyltransferase	10	IU/L
Total protein	72	g/L
Albumin	34	g/L
Blood urea nitrogen	23.6	mmol/L
Uric acid	434	μmol/L
Creatinine	910	μmol/L
Sodium	136	mmol/L
Potassium	6.6	mmol/L
Chloride	97	mmol/L
Calcium	2.22	mmol/L
Phosphorus	2.42	mmol/L
Magnesium	0.99	mmol/L
C-reactive protein	2000	μg/L
Intact parathyroid hormone	707,000	μg/L
Bone type alkaline phosphatase	18.6	IU/L
Tartrate-resistant acid phosphatase 5b	818	mU/dL

We resected one large nodule and three normal-size parathyroid glands. As the largest gland had macroscopically invaded the left sympathetic nerve and clearly invaded muscle layer of the esophagus, we employed an en bloc excision. Histopathological analysis revealed that the parathyroid cells had contiguously invaded beyond the fibrous capsules into the surrounding skeletal muscle tissue (Fig. 1d). Thus, a diagnosis of parathyroid carcinoma was made.

Two weeks after the surgery, the patient’s pain while walking had disappeared. To prevent postoperative hungry bone syndrome, calcium carbonate and alfacalcidol were administered for 2 months and then tapered off. X-ray films showed a significant decrease in the tumoral calcinosis of the right hip joint and left knee after 3 months (Fig. 1b). Her chest pain during mild exercise disappeared. Her phosphate level was controlled below 1.94 mmol/L with a non-calcium-containing phosphate binder and longer dialysis time (4.5–5.0 h). Her iPTH level was 0 to 2000 μg/L under a relatively low calcium level (2.00–2.12 mmol/L), indicating that a total PTx was successfully achieved in this case.

## Discussion

This case clearly indicated that the use of cinacalcet hinders the diagnosis of parathyroid carcinoma in a chronic dialysis patient. Cinacalcet is a calcimimetic drug that reduces PTH secretion by increasing the sensitivity of the calcium-sensing receptor (Ca-SR), which is specifically expressed in parathyroid cells [10, 11]. Cinacalcet affects parathyroid adenoma and carcinoma, which express Ca-SR on the cell surface, decreases the Ca level, and suppresses tumor growth [9, 12, 13]. Although cinacalcet is used extensively in the treatment of SHPT in chronic dialysis patients worldwide, clinical trials have not determined if cinacalcet would reduce cardiovascular events and improve mortality [14, 15].

Parathyroid carcinoma accounts for between 0.5% and 5% of all cases of primary hyperthyroidism [16, 17]. Approximately 700 parathyroid carcinoma cases have been reported, 20 of which occurred in chronic dialysis patients [16]. A Japanese retrospective study reported 3.14% parathyroid carcinomas in 826 parathyroidectomies [18]. As it is difficult to distinguish parathyroid carcinoma from parathyroid hyperplasia (which is the most common cause of SHPT) by routine image diagnosis, cinacalcet might cause a delay in the diagnosis of parathyroid carcinoma when it is used to control the PTH level in chronic dialysis patients.

Classically, the clinical features of parathyroid carcinoma include hypercalcemia, palpable neck mass, high serum PTH, and osteitis fibrosa cystica [19–21]. The principle histological features were reported by Castleman et al. to be: 1) a trabecular pattern, 2) mitotic figures, 3) thick fibrous bands, and 4) capsular and blood vessel invasion [22]. Since these features have also occasionally been noted in parathyroid adenoma and hyperplasia, two criteria were added later; the first is the local invasion of contiguous structures, and the second is lymph node or distant metastasis [23]. Our case did not have the typical clinical features described above, although the parathyroid cells invaded beyond the fibrous capsules pathologically and spread into the surrounding muscle also macroscopically, resulting in tight adhesion. Indeed, we had to perform an en bloc excision to resect the tumor during surgery. As the patient had not undergone treatment with other interventions for her parathyroid gland (such as percutaneous ethanol injection therapy), which could be a cause of secondary parathyromatosis, the surgical and histological findings strongly support the diagnosis of the largest parathyroid gland with carcinoma [24–26]. The recurrence rate for parathyroid carcinoma is reported to be 27, 82, and 91% after 1, 5, and 10 years, respectively [27]. Thus, the iPTH level should be monitored in order to ensure early detection of a recurrence.

A non-calcium-containing phosphate binder can suppress the progression of ectopic calcification, such as vascular calcification and tumorous calcinosis [5]. The combination of cinacalcet with Vitamin D might, however, lead to a decrease in bone turnover as a result of the decrease in PTH. Under these conditions, hypercalcemia with hyperphosphatemia due to the decreased buffering effect of bone could result in a positive load of calcium and phosphate into the soft tissue, resulting in ectopic calcification [28]. PTx can trigger hungry bone syndrome, which accelerates the draw of calcium and phosphate from the lesions of ectopic calcification sites and squeezes them into the bone [4].

In our case, the PTx allowed us to both treat the ectopic calcification and diagnose it as parathyroid carcinoma, and the serum calcium and PTH dropped when parathyroid carcinoma was removed. The iPTH value of SHPT patients who should be treated with PTx is recommended as 500,000 μg/L by the Japanese guideline [7]. Most symptomatic patients who receive chronic dialysis and who undergo PTx have a serum iPTH level of more than 800,000 μg/L [29]. Asymptomatic patients are, however, commonly referred for parathyroidectomy when they have a sustained PTH of more than 1000,000 μg/L [7, 30]. Indeed, cinacalcet came on the market in Japan in 2008, and the number of PTx in Japan decreased strikingly as a result [15, 18, 31]. Earlier PTx should be considered before a vicious exacerbation of ectopic calcification. Further study is required to determine the new indications of PTx in patients receiving chronic dialysis therapy and cinacalcet treatment.

## Conclusions

We successfully treated a chronic dialysis patient for parathyroid carcinoma with tumoral calcinosis by total PTx. The use of cinacalcet hinders the diagnosis of parathyroid carcinoma in a chronic dialysis patient.

## Abbreviations

ALP: alkaline phosphatase
Ca: calcium
Ca-SR: calcium-sensing receptor
iP: inorganic phosphate
iPTH: intact parathyroid hormone
PTx: parathyroidectomy
SHPT: secondary hyperparathyroidism
